# Why *one-size-fits-all* vaso-modulatory interventions fail to control glioma invasion: *in silico* insights

**DOI:** 10.1038/srep37283

**Published:** 2016-11-23

**Authors:** J. C. L. Alfonso, A. Köhn-Luque, T. Stylianopoulos, F. Feuerhake, A. Deutsch, H. Hatzikirou

**Affiliations:** 1 Braunschweig Integrated Centre of Systems Biology and Helmholtz Center for Infectious Research, Braunschweig, Germany; 2 Center for Information Services and High Performance Computing, Technische Universität Dresden, Germany; 3 Department of Biostatistics, Faculty of Medicine, University of Oslo, Norway; 4 BigInsight, Centre for Research-based Innovation (SFI), Oslo, Norway; 5 Cancer Biophysics Laboratory, Department of Mechanical and Manufacturing Engineering, University of Cyprus, Nicosia, Cyprus; 6 Institute of Pathology, Medical School of Hannover, Germany; 7 Institute of Neuropathology, University Clinic Freiburg, Germany

## Abstract

Gliomas are highly invasive brain tumours characterised by poor prognosis and limited response to therapy. There is an ongoing debate on the therapeutic potential of vaso-modulatory interventions against glioma invasion. Prominent vasculature-targeting therapies involve tumour blood vessel deterioration and normalisation. The former aims at tumour infarction and nutrient deprivation induced by blood vessel occlusion/collapse. In contrast, the therapeutic intention of normalising the abnormal tumour vasculature is to improve the efficacy of conventional treatment modalities. Although these strategies have shown therapeutic potential, it remains unclear why they both often fail to control glioma growth. To shed some light on this issue, we propose a mathematical model based on the migration/proliferation dichotomy of glioma cells in order to investigate why vaso-modulatory interventions have shown limited success in terms of tumour clearance. We found the existence of a critical cell proliferation/diffusion ratio that separates glioma responses to vaso-modulatory interventions into two distinct regimes. While for tumours, belonging to one regime, vascular modulations reduce the front speed and increase the infiltration width, for those in the other regime, the invasion speed increases and infiltration width decreases. We discuss how these *in silico* findings can be used to guide individualised vaso-modulatory approaches to improve treatment success rates.

Gliomas are aggressive brain tumours typically associated with poor prognosis, sharp deterioration in the patients’ quality of life and low survival rates, making this disease challenging to treat. According to the World Health Organization (WHO)^1^, gliomas are classified into different categories varying from low-grade (slowly-growing) to high-grade (rapidly-growing) tumours depending on their proliferative capacity and invasiveness, with glioblastoma multiforme (GBM) being the most malignant form. Despite significant advances in surgical and medical neuro-oncology^2^,^3^, complete tumour resection is unlikely and subsequent recurrence is almost inevitable. A major obstacle to cure this devastating type of brain tumours is attributed to its highly invasive nature. In fact, glioma cells have a remarkable ability to infiltrate the surrounding brain tissue and migrate long distances away from the tumour bed, which enables them to escape surgical resection, radiation exposure and chemotherapy^4^,^5^,^6^. The persistently poor prognosis, together with the high treatment failure rates demand more effective therapeutic strategies that should be based on a deeper mechanistic understanding of the key events triggering glioma invasion.

The influence of the microenvironment on glioma cell behaviour plays a crucial role in the resulting diffusive tumour growth and its invasive capacity. Hypoxia, the presence of abnormal and sustained low oxygen levels in the tumour tissue, strongly correlates with glioma invasiveness and malignancy^7^. At high glioma cell density, tumours contain hypoxic regions with an inadequate oxygen supply due to tumour-induced vascular abnormalities. Under such oxygen-limiting conditions, glioma cells develop a wide variety of rescue mechanisms to survive and sustain proliferation. These include recruitment of new blood vessels driven by secretion of pro-angiogenic factors, modulation of cell oxygen consumption and activation of cell migration to escape from poorly oxygenated regions^8^,^9^,^10^,^11^. In particular, the ability of glioma cells to switch phenotype in response to metabolic stress is believed to have important implications for tumour progression and resistance to therapeutic agents. For instance, the mutually exclusive switching between proliferative and migratory phenotypes experimentally observed, also known as the migration/proliferation dichotomy or Go-or-Grow mechanism, is considered to significantly increase the invasive potential of glioma cells in response to low oxygen levels^4^,^10^,^12^,^13^,^14^. However, the way in which the dynamical interplay between glioma cells and their microenvironment leads to development of hypoxic regions, as well as the overall impact of oxygen availability on tumour invasion are still not fully understood.

A particularly important component of the tumour microenvironment is the vascular network. Accumulating evidence suggests the existence of various positive and negative feedback mechanisms between glioma cells and the vasculature. Indeed, gliomas are reported as highly vascularised neoplasias^15^,^16^, where excessive blood vessel formation is induced by a wide range of pro-angiogenic factors^17^,^18^. However, over-expression of pro-angiogenic factors produced by hypoxic glioma cells is commonly observed, which ultimately results in local vascular hyperplasia and focal areas of necrosis. Such functional and morphological abnormalities in the tumour-associated vasculature are common features of gliomas, with blood vessels of significantly larger diameters, higher permeability and thicker basement membranes than those found in the normal brain tissue^15^, see Fig. 1(A,B). Moreover, blood vessel occlusion has been reported to initiate a hypoxia/necrosis cycle influencing the dynamical balance between glioma cell migration and proliferation. In fact, several clinical and experimental observations suggest that vaso-occlusion could readily explain the rapid peripheral expansion and invasive behaviour of gliomas^19^,^20^. Vaso-occlusion can mainly occur due to increased mechanical pressure exerted on the blood vessels by tumour cells or induced by intravascular pro-thrombotic mechanisms^21^,^22^, see Fig. 1(C,D). Occluded or collapsed blood vessels could lead to perivascular hypoxia, necrosis and hypercellular zones referred to as pseudopalisades, which induce collective cell migration. Actually, these vascular occlusive events have been linked to waves of hypoxic glioma cells actively migrating away from oxygen-deficient and necrotic regions^19^,^20^,^21^,^23^. Since hypoxia-induced migration has been long recognised to support further glioma cell invasion, it may be crucial to investigate the overall effect of vaso-modulatory interventions on the tumour front speed and infiltration width.

The high degree of angiogenesis and vascular pathologies observed in gliomas has been the target of several therapeutic vaso-modulatory strategies^24^,^25^. Clinical and preclinical findings suggest that angiogenesis inhibitors alone, with the potential to starve glioma cells, have limited efficacy in terms of tumour shrinkage, functional vasculature destruction and patient survival^26^,^27^,^28^. Furthermore, anti-angiogenic factors as inhibitors of neovascularisation are also restricted by transient effects and development of therapy resistance^29^. Instead, improved tumour vascularisation, either via normalisation or through a stress alleviation strategy based on reopening compressed blood vessels, is an emerging concept expected to reduce tumour hypoxia, improve perfusion, enhance the delivery of cytotoxic drugs and increase radiotherapy efficacy^24^,^30^,^31^,^32^. Interestingly, recent evidence reveals that judicious application of an anti-angiogenic therapy may normalise the structure and function of the tumour vasculature^28^,^30^,^31^, where the success rate is schedule- and patient-dependent^33^,^34^. Although vasculature-targeting interventions could provide therapeutic benefits, further mechanistic insights into their influence on glioma cell dynamics are still needed to improve treatment outcomes^24^,^32^.

Mathematical modelling has the potential to improve our understanding of the complex biology of gliomas and their interactions with the microenvironment, as well as it may help in the design of more effective and personalised treatment strategies^35^,^36^,^37^,^38^,^39^,^40^,^41^,^42^,^43^. Several mathematical models have been developed to identify mechanisms and factors that facilitate proliferation and migration of glioma cells^16^,^38^,^44^,^45^,^46^,^47^,^48^,^49^,^50^,^51^,^52^,^53^, as well as to explore processes related to malignant progression^54^,^55^,^56^. Most of these models have been formulated to examine glioma growth and invasion based exclusively on cellular diffusion and proliferation rates^44^,^45^,^46^,^47^,^49^. Recently, models including the influence of different tumour microenvironmental factors such as hypoxia, necrosis and angiogenesis have been also proposed^16^,^38^,^53^. However, the impact of vascular occlusive events or vascular normalisation on glioma invasion, considering the Go-or-Grow mechanism, has not been addressed so far. In this work, we propose a mathematical model to investigate the reasons for which vaso-modulatory interventions often fail to control glioma invasion. In particular, we focus on the interplay between the migration/proliferation dichotomy of glioma cells and variations in the functional tumour vasculature. The aim is to generate novel insights into the impact of vaso-modulatory interventions on tumour front speed and infiltration width, as well as to discuss the therapeutic potential of a combination of vasculature-targeting strategies with other treatment protocols for personalized medicine. We begin by defining the biological assumptions taken into account when developing our glioma-vasculature interplay model. Then, we study the effects of modulations of cell oxygen consumption and vaso-occlusion rates on glioma invasion. We show that one-size-fits-all vaso-modulatory interventions should be expected to fail to control glioma invasion, since there is a trade-off between tumour front speed and infiltration width. The model provides a better understanding of glioma-microenvironment interactions and is suited for analysing the potential success or failure of vaso-modulatory treatments. We conclude by discussing the main implications of our model in the design of novel approaches for individualised therapy.

## Methods

### The glioma-vasculature interplay model

We develop a mathematical model that describes the growth of vascularised gliomas focusing on the interplay between the migration/proliferation dichotomy and vaso-occlusion at the margin of viable tumour tissue. The system variables are the density of glioma cells *ρ*(*x*, *t*) and functional tumour vasculature *v*(*x*, *t*), as well as the concentrations of oxygen *σ*(*x*, *t*) and pro-angiogenic factors *a*(*x*, *t*) in the tumour microenvironment, where 
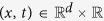
 and *d* is the dimension of the system. Figure 2(A) shows a schematic representation of the system interactions and model assumptions, which are summarised as follows:

**[A1]** Glioma cells switch phenotypes between proliferative (normoxic) and migratory (hypoxic) depending on the oxygen concentration in the tumour microenvironment^4^,^10^,^12^,^13^,^14^.

**[A2]** Under hypoxia conditions glioma cells secrete pro-angiogenic factors^15^,^17^,^18^,^24^.

**[A3]** Pro-angiogenic factors trigger the formation of blood vessels and regulate vasculature remodelling^18^,^28^.

**[A4]** Endothelial cells forming the vascular network uptake pro-angiogenic factors^18^,^57^.

**[A5]** Functional tumour blood vessels releases oxygen^15^,^17^,^18^.

**[A6]** Oxygen is essential for glioma growth and progression^17^,^18^,^58^.

**[A7]** Glioma cells consume oxygen provided by the functional vasculature^17^,^59^.

**[A8]** Prothrombotic factors and increased mechanical pressure in regions of high glioma cell density induce blood vessel occlusion and collapse^19^,^23^,^58^,^60^.

#### Density of glioma cells, *ρ*(*x*, *t*)

Based on the migration/proliferation dichotomy^4^,^10^,^12^,^13^,^14^, we assume that glioma cells switch between two different phenotypes, migratory (hypoxic) *ρ*_1_(*x*, *t*) and proliferative (normoxic) *ρ*_2_(*x*, *t*), depending on the concentration of oxygen in the tumour microenvironment *σ*(*x*, *t*). More precisely, we consider two linear switching functions, *f*_21_(*σ*) = *λ*_1_ − *σ* and *f*_12_(*σ*) = *λ*_2_*σ*, that represent the rate at which glioma cells change from migratory to proliferative and vice versa, respectively. Although there is experimental evidence of a positive correlation between oxygen availability and cell proliferation, the exact functional form of the oxygen-dependent phenotypic switching remains unknown. Accordingly, we consider the simplest case, i.e. a linear switching between proliferative and migratory phenotypes, in line with previous studies^61^,^62^. The parameters *λ*_1_ and *λ*_2_ are positive constants, see the Supplementary Material for further details.

Cell motility is modelled as a diffusive process mimicking the net infiltration of glioma cells into the surrounding brain tissue, while a logistic growth term is considered for tumour cell proliferation. The system of equations for the migratory and proliferative glioma cells is given by


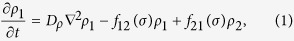



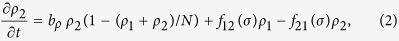


where the temporal *t* and spatial *x* coordinates in the arguments of variables have been omitted for notational simplicity. *D*_*ρ*_ and *b*_*ρ*_ are the diffusion and proliferation coefficients of migratory and proliferative glioma cells, respectively. *N* represents the brain tissue carrying capacity, i.e. the maximum number of cells that can be supported by the environment. The parameters *D*_*ρ*_, *b*_*ρ*_ and *N* are positive constants.

The system (1)-(2) can be reduced to a single equation for the total density of glioma cells *ρ* = *ρ*_1_ + *ρ*_2_ by assuming that *f*_12_(*σ*)*ρ*_1_ = *f*_21_(*σ*)*ρ*_2_. This is a plausible assumption since intracellular processes, such as signalling pathways regulating the phenotypic switch, operate at much shorter time scales than cell migration and proliferation. Thus, we assume that phenotype switching is a mechanism faster compared to cell division and motility, which allows to express *ρ*_1_ and *ρ*_2_ as a function of *ρ* in the following form


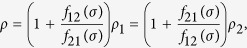


where we have that


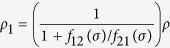


and


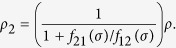


Summing equations (1) and (2), and substituting the expressions above for *ρ*_1_ and *ρ*_2_, the equation for the total density of (migratory and proliferative) glioma cells *ρ*(*x*, *t*) is given by


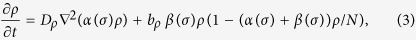


where the oxygen-dependent functions *α*(*σ*) and *β*(*σ*) are defined as follows


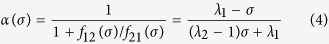


and


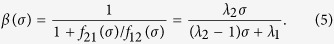


Then, taking into account that *α*(*σ*) + *β*(*σ*) = 1, we can rewrite equation (3) in the following form


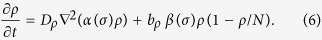


Notice that equation (6) is a generalisation of the widely studied Fisher-Kolmogorov model which describes glioma growth and invasion^55^,^63^. The nonlinear terms *α*(*σ*) and *β*(*σ*) in equation (6) modulate the rates of glioma cell diffusion and proliferation according to oxygen availability. Under hypoxic conditions cell diffusion increases, while proliferation decreases, i.e. glioma cells become more migratory and less proliferative. On the contrary, at normal oxygen levels (normoxic conditions) glioma cells become more proliferative and less migratory. Let *σ*_0_ > 0 be the physiological oxygen concentration in the normal brain tissue. Then, by normalising *D*_*ρ*_ = *D*/*α*(*σ*_0_) and *b*_*ρ*_ = *b*/*β*(*σ*_0_) the classical Fisher-Kolmogorov equation is recovered under the assumption of a constant oxygen concentration in the tumour microenvironment, given by


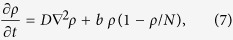


where *D* and *b* are positive parameters that represents the intrinsic diffusion and proliferation rates of glioma cells, respectively. We remark that, equation (7) has been extensively used to predict untreated glioma kinetics based on patient-specific parameters from standard medical imaging procedures^16^,^49^,^55^,^64^. Furthermore, the Fisher-Kolmogorov equation has been also considered to estimate glioma recurrence after surgical resection^50^ and simulate of tumour responses to conventional therapeutic modalities such as chemo-48 and radiotherapy^65^.

#### Pro-angiogenic factor concentration, *a*(*x*, *t*)

Neovascularisation in tumours takes place when pro-angiogenic factors overcome anti-angiogenic stimuli. However, in gliomas there is evidence of a wide range of pro- and anti-angiogenic factors involved, each of them acting through different vascularisation mechanisms^15^,^24^,^28^. While not explicitly considering the vascular endothelial growth factor (VEGF) or any other specific pro-angiogenic chemokine, we assume a generic effective pro-angiogenic factor concentration at quasi-steady state. In fact, we suppose that an over-expression of pro-angiogenic factors instantaneously promotes the formation of functional tumour vasculature *v*(*x*, *t*). We further consider that pro-angiogenic factors are exclusively produced by glioma cells under hypoxic conditions at a rate proportional to the tumour cell density, and therefore neglecting hypoxia-independent pathways. In addition, endothelial cells forming the vascular network uptake pro-angiogenic factors which also undergo natural decay. The equation for the effective pro-angiogenic factor concentration *a*(*x*, *t*) is given by


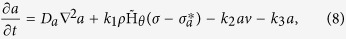


where the temporal *t* and spatial *x* coordinates in the arguments of variables have been omitted for notational simplicity. *D*_*a*_ is the diffusion coefficient of pro-angiogenic factors. Assuming the quasi-steady state approximation of equation (8), we have that


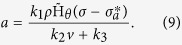


The positive parameters *k*_1_, *k*_2_ and *k*_3_ represent the production, consumption and natural decay rates of pro-angiogenic factors, respectively, where 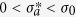 is the hypoxic oxygen threshold for their production by glioma cells. 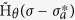 is a continuous approximation of the Heaviside decreasing step function *H*(*ξ*), defined as *H*(*ξ*) = 1 if *ξ* ≤ 0 and *H*(*ξ*) = 0 if *ξ* > 0, given by


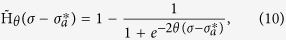


where *θ* is a positive parameter that controls the steepness of 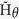 at 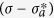. More precisely, 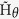 models the production of pro-angiogenic factors by glioma cells when the oxygen concentration *σ* is lower than the hypoxic oxygen threshold 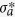.

#### Density of functional tumour vasculature, *v*(*x*, *t*)

**Figure 1:**
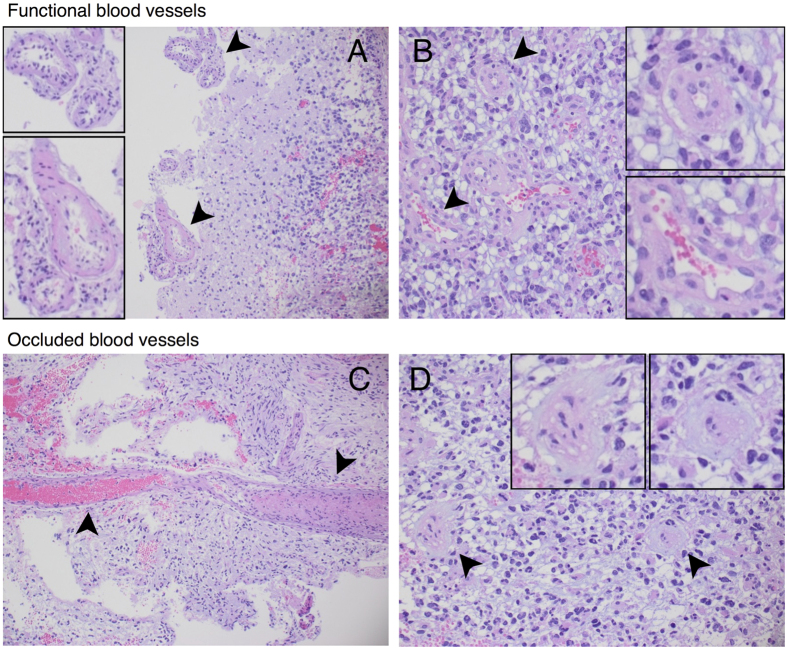
Histological images of functional and occluded blood vessels in gliomas. (**A**) From right to left brain tissue infiltrated by glioma cells with meningeal blood vessels of normal size and anatomy. (**B**) Atypical but not occluded intratumoural blood vessels with activated endothelium and thicker/plumper muscular layers than the normal brain blood vessels. (**C**) A longitudinal section of a large intratumoural blood vessel with a no-obliterated part filled with blood (left) and an occluded thrombotic part (right). (**D**) Thrombotic occlusion in small intratumoural blood vessels. The arrowheads point to blood vessels which are magnified in the corresponding subfigures.

Histopathological studies have shown that the vascular structure and function in brain tumours is markedly abnormal^17^,^18^,^58^. Gliomas, and particularly glioblastomas, are known to have blood vessels of increased diameter, high permeability, thickened basement membranes and highly proliferative endothelial cells^15^, see Fig. 1(B). Due to such abnormalities, a significant fraction of the tumour-associated vasculature does not constitute functional blood vessels^15^. Based on these facts, we only consider functional vascularisation instead of modelling the complete tumour vascular network. Accordingly, we assume that the density of functional tumour vasculature is a dimensionless and normalised quantity with values in the interval [0, 1]. The normal density of functional blood vessels in the normal brain tissue is taken as *v* = 1/2. Thus, the limit case *v* = 0 represents an avascular tissue, while on the contrary *v* = 1 describes a hypothetical scenario characterised by excessive vascularisation.

Blood vessels in gliomas are not stable, being continuously formed, occluded and destroyed. Neovascularisation takes place by different angiogenic and vasculogenic processes induced by complex signalling mechanisms that are not well understood^11^,^66^,^67^. For simplicity, we assume that tumour blood vessels are created when pro-angiogenic factors prevail anti-angiogenic stimuli, i.e. for *a* > 0, leading to the formation of new functional vasculature according to a logistic growth term. The rate at which functional tumour vasculature is generated follows the Michaelis-Menten kinetics depending on the pro-angiogenic factor concentration, where a constant dispersal rate of endothelial cells (vasculature) is assumed. Notice that the Michaelis-Menten term is commonly used to model a saturating response at high doses in biological systems^22^,^63^,^68^. On the other hand, we consider that mechanical or chemical cues in regions of high glioma cell density induce blood vessel occlusion or collapse^19^,^23^,^60^. Vaso-occlusion is then modelled by a power law dependence on the density of glioma cells. The equation for the density of functional tumour vasculature *v*(*x*, *t*) is given by


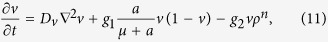


where again the temporal *t* and spatial *x* coordinates in the arguments of variables have been omitted for notational simplicity. *D*_*v*_ is the diffusion coefficient representing the net dispersal of tumour vasculature, *g*_1_ is the formation rate of functional blood vessels, *μ* is the pro-angiogenic factor concentration at which *g*_1_ is half-maximal, *g*_2_ is the vaso-occlusion rate and *n* is a parameter that regulates the degree of blood vessel occlusion depending on the density of glioma cells. The vaso-occlusion term, *g*_2_*vρ*^*n*^, models the mechanical pressure exerted on blood vessels in regions of high glioma cell density, see the Supplementary Material for further details. When the intratumoural cellular pressure exceeds a critical threshold, massive tumour blood vessel collapse occurs^32^,^69^. However, prior to this critical stress threshold, blood vessel collapse is moderate^69^. In particular, we assume that vaso-occlusion only occurs for glioma cell densities greater than *N*/2, where *N* is the brain tissue carrying capacity^60^. The parameters *D*_*v*_, *g*_1_, *μ*, *g*_2_ and *n* are positive constants.

Plugging equation (9) for the effective pro-angiogenic factor concentration into equation (11), and assuming that the decay rate of *a* is much smaller than the uptake/internalisation rate by endothelial cells, i.e. *k*_3_ ≪ *k*_2_^70^,^71^, we have that


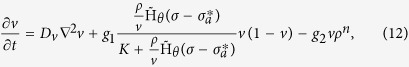


where *K* = *μk*_2_/*k*_1_ represents the concentration of pro-angiogenic factors at which the formation rate of functional tumour vasculature is half-maximal, see the Supplementary Material for more details.

#### Oxygen concentration, *σ*(*x*, *t*)

Oxygen is delivered to the brain tissue via functional blood vessels, spreads into the tumour bulk and is consumed by glioma cells. Transport of oxygen within tissues occurs by diffusion and convection^72^. For simplicity, we neglect the convective contribution and only consider that after transvascular exchange oxygen molecules move exclusively by diffusion. The delivery of oxygen to the tumour is modelled by assuming that the supply rate is proportional to the functional vasculature and the difference between the physiological oxygen concentration in the normal brain tissue *σ*_0_ and that in the tumour interstitium. These assumptions result in the equation for the oxygen concentration *σ*(*x*, *t*) given by


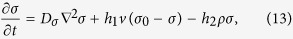


where the temporal *t* and spatial *x* coordinates in the arguments of variables have been omitted for notational simplicity. *D*_*σ*_ is the oxygen diffusion coefficient, *h*_1_ is the permeability coefficient of functional vasculature and *h*_2_ is the oxygen consumption rate by glioma cells. The parameters *D*_*σ*_, *h*_1_, *σ*_0_ and *h*_2_ are positive constants. Notice that similar assumptions have been previously considered to model oxygen dynamics in vascular tumour growth^22^.

#### Model formulation, boundary and initial conditions

The proposed glioma-vasculature interplay model comprises a system of coupled partial differential equations given by


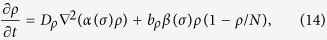



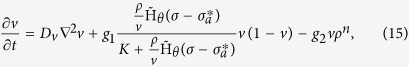



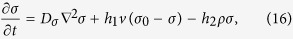


where the oxygen-dependent functions *α*(*σ*) and *β*(*σ*) are given by equations (4)–(5), respectively. The system (14)–(16) is closed by imposing the following initial conditions


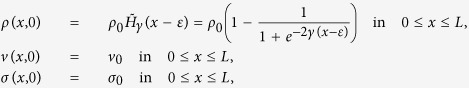


where the positive parameters *ρ*_0_, *σ*_0_ and *v*_0_ are the initial density of glioma cells spatially distributed in a segment of length *ε*, the density of functional tumour vasculature and the oxygen concentration, respectively. The positive parameter *γ* controls the steepness of 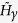 at (*x* − *ε*) with *ε* > 0, and *L* > 0 is the length of the one-dimensional computational domain. In addition, we consider an isolated host tissue in which all system behaviours arise solely due to the interaction terms in equations (14)–(16), 25, 26. This assumption results in no-flux boundary conditions of the form


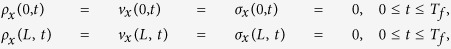


where *T*_*f*_ > 0 is an arbitrary simulation time, i.e. the end of simulations. The conditions above also imply that no cell or molecule leaves the system through the domain boundaries.

### Modelling hierarchy

**Figure 2:**
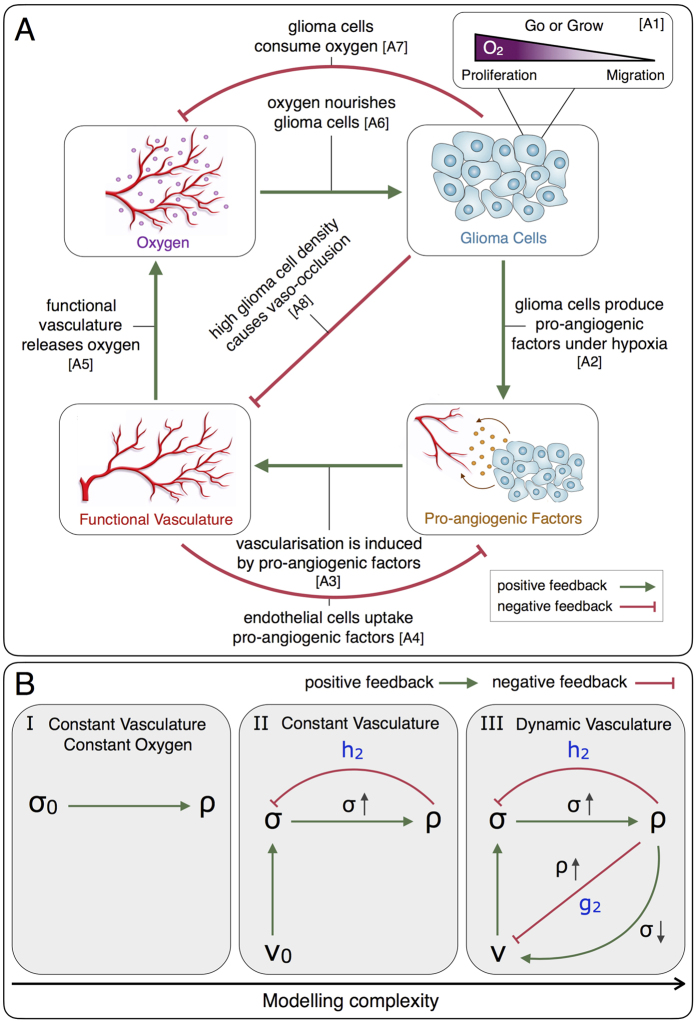
Modelling logic and hierarchy. (**A**) Diagram of the interactions between glioma cells, oxygen availability, functional tumour vasculature and pro-angiogenic factors. (**B**) From left to right model complexity increases with respect to the interactions between system variables: density of glioma cells *ρ*(*x*, *t*), density of functional tumour vasculature *v*(*x*, *t*) and oxygen concentration *σ*(*x*, *t*). The parameters *σ*_0_ and *v*_0_ represent constant oxygen concentration and functional tumour vascularisation, respectively. The parameters *h*_2_ and *g*_2_ are the glioma cell oxygen consumption and vaso-occlusion rates, respectively (see equations (12)–(13).

The glioma-vasculature interplay model (14)–(16) referred to as *Model III*, is a generalisation of two simpler models which are also of interest for the study of glioma growth and invasion. As shown in Fig. 2(B), such simpler models can be obtained under the assumptions of a constant density of functional tumour vasculature *v*(*x*, *t*) = *v*_0_ (*Model II*), and also a constant oxygen concentration *σ*(*x*, *t*) = *σ*_0_ (*Model I*). More precisely, *Model II* is obtained from *Model III* by setting *g*_1_ = *g*_2_ = 0 in equation (15), i.e. assuming neither formation nor occlusion/collapse of tumour blood vessels. In turn, *Model I* is obtained from *Model II* by setting *h*_2_ = 0 in equation (16), i.e. assuming a constant oxygen concentration in the tumour microenvironment.

*Model I* corresponds to the classical Fisher-Kolmogorov equation (7), for which a large number of theoretical and simulation results have been reported^55^,^63^. *Model II* given by equations (14) and (16) contains an extended version of the Fisher-Kolmogorov equation with nonlinear glioma cell diffusion and proliferation terms. Both nonlinearities depend on the oxygen concentration in the tumour microenvironment, which is governed by a reaction-diffusion equation with linear diffusion and nonlinear reaction terms. Notice that reaction-diffusion is a process in which more than one component, i.e. chemical species and/or population of cells, are assumed to diffuse over a surface and react with each other. In addition, the dynamic of glioma cells is modelled by considering the migration/proliferation dichotomy (Go-or-Grow mechanisms). Since the supply of oxygen rate in *Model II* is assumed constant, the blood perfusion can be considered stable and we therefore neglect tumour-induced vascular pathologies. The latter is a reasonable assumption, particularly for low-grade gliomas, where an abnormal vascular structure is not prominent^16^. A natural extension of *Model II* is to consider tumour-associated vascularisation dynamics. This is precisely what defines *Model III*, which is used to investigate the effects of vaso-modulatory interventions on glioma invasion. Taking into account that *Model I* has been extensively studied, we begin with the analysis of *Model II* as an intermediate step towards analysing *Model III*, see Fig. 2(B). In particular, we focus on the effects of variations in the glioma cell oxygen consumption and vaso-occlusion rates on tumour front speed and infiltration width. In the Supplementary Material we provide details about the numerical implementation of the model, as well as additional simulation results.

### Model observables

We characterise glioma invasion by the tumour front speed and infiltration width, see Figure S1 in the Supplementary Material. The tumour front speed is estimated by the change rate of the point of maximum slope in *ρ*(*x*, *t*) at the end of simulations *T*_*f*_. In turn, the infiltration width is defined by the difference between the points where glioma cell density is 80% and 2% of the maximum cellular density at simulation time *T*_*f*_. These specific features of tumour invasion have been reported crucial to determine glioma malignancy and predict therapeutic failure^16^,^50^,^55^.

Unlike the classical Fisher-Kolmogorov equation (7), in our glioma-vasculature interplay invasion model (14)–(16) cellular processes are regulated by oxygen availability. Therefore, we distinguish the intrinsic glioma cell diffusion *D* and proliferation *b* rates from the effective rates that depend on the oxygen concentration in the tumour microenvironment. The effective diffusion *D*_eff_ and proliferation *b*_eff_ rates of glioma cells are defined as follows


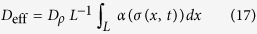


and


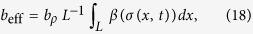


where *L* is the length of the one-dimensional domain of simulation. Notice that *D*_*ρ*_ = *D*/*α*(*σ*_0_) and *b*_*ρ*_ = *b*/*β*(*σ*_0_), where *D* and *b* are the intrinsic glioma cell diffusion and proliferation rates, respectively. We then investigate the dependence of *D*_eff_ and *b*_eff_, as well as the tumour front speed and infiltration width, at simulation time *T*_*f*_ on different values of parameters *h*_2_ (glioma cell oxygen consumption) and *g*_2_ (vaso-occlusion).

### Model parameterisation

Parameter values considered in the model simulations are taken from published data wherever possible or estimated to approximate physiologic conditions based on appropriate physical and biological arguments, see Table 1 and the Supplementary Material for more details. For parameters of special interest, a wide range of values is considered to explore their effects on glioma growth and invasion.

## Results

### Increasing oxygen consumption and vaso-occlusion results in more diffusive and less proliferative gliomas

**Figure 3:**
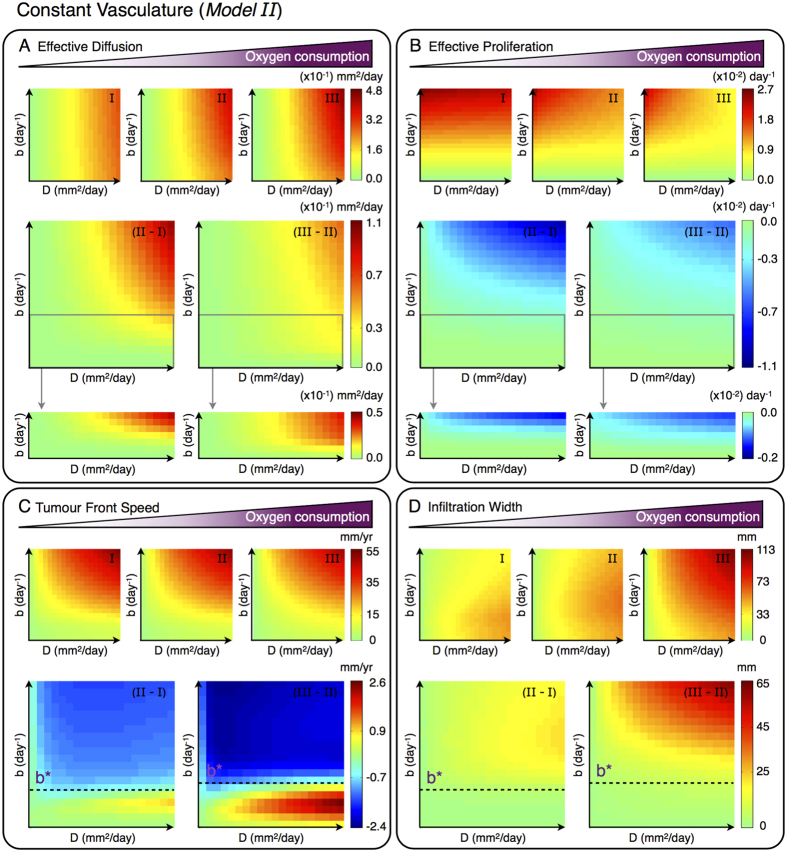
Glioma cell oxygen consumption effects on tumour invasion for constant functional vascularisation (*Model II*). Simulation maps with respect to the intrinsic proliferation *b* ∈ [2.73 × 10^−4^, 2.73 × 10^−2^] days^−1^ and diffusion *D* ∈ [2.73 × 10^−3^, 2.73 × 10^−1^] mm^2^ days^−1^ rates of glioma cells. (**A**) Effective diffusion, (**B**) effective proliferation, (**C**) tumour front speed and (**D**) infiltration width for different glioma cell oxygen consumption rates *h*_2_ = {5.73 × 10^−4^, 5.73 × 10^−3^, 5.73 × 10^−2^} mm cell^−1^ day^−1^ in simulation maps I-III, respectively. (**A–D**) Differences between the simulation maps I-III. The other parameters are as in Table 1.

**Figure 4:**
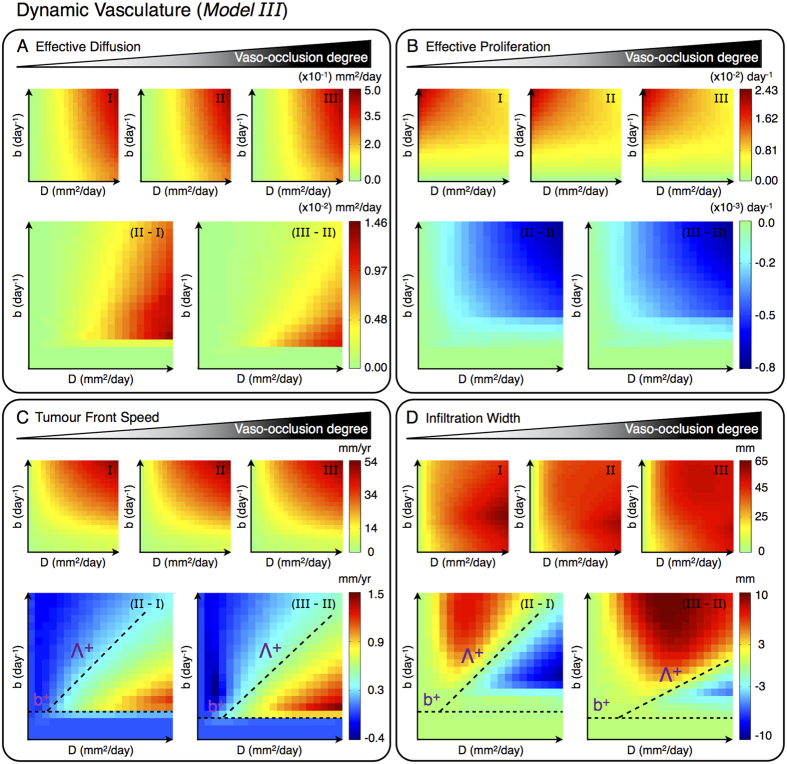
Vaso-occlusion effects on glioma invasion (*Model III*). Simulation maps with respect to the intrinsic proliferation *b* ∈ [2.73 × 10^−4^, 2.73 × 10^−2^] days^−1^ and diffusion *D* ∈ [2.73 × 10^−3^, 2.73 × 10^−1^] mm^2^ days^−1^ rates of glioma cells. (**A**) Effective diffusion, (**B**) effective proliferation, (**C**) tumour front speed and (**D**) infiltration width for a constant glioma cell oxygen consumption rate *h*_2_ = 5.73 × 10^−3^ mm cell^−1^ day^−1^ and different vaso-occlusion rates *g*_2_ = {5.0 × 10^−13^, 5.0 × 10^−12^, 1.5 × 10^−11^} cells^−*n*^ mm^*n*^ day^−1^ in simulation maps I-III, respectively. (**A–D**) Differences between the simulation maps I-III. The other parameters are as in Table 1.

The proposed glioma-vasculature interplay model (14)–(16) is first considered to investigate the effects of variations in the tumour cell oxygen consumption and vaso-occlusion rates on the effective invasive behaviour of gliomas. Figures 3(A,B) and 4(A,B) show simulation maps of the effective diffusion *D*_eff_ and proliferation *b*_eff_ rates defined in equations (17) and (18) respectively, for tumours characterised by different combinations of the intrinsic glioma cell features *D* and *b*. Model simulations in Fig. 3(A,B) are obtained under the assumption of a constant density of functional tumour vasculature, i.e. neither formation nor occlusion/collapse of tumour blood vessels, for increasing oxygen consumption rates by glioma cells. In turn, Fig. 4(A,B) shows simulation maps for a constant rate of oxygen consumption by tumour cells, considering tumour vascularisation dynamics and increasing vascular occlusive events.

Comparative simulation maps in Figs 3(A,B) and 4(A,B) illustrate that an arbitrary increase in either the rate at which glioma cells consume oxygen *h*_2_ or vaso-occlusion *g*_2_ results in more diffusive and less proliferative tumours. The model supports that at high *h*_2_ and *g*_2_ values, the oxygen concentration in the tumour microenvironment significantly decreases, which may result in hypoxia and necrosis. The lack of oxygen limits the proliferative capacity of glioma cells, and in turn enhances the hypoxia-induced cell migration to better-oxygenated brain tissue areas. In particular, variations in the cell oxygen consumption and vaso-occlusion rates are predicted to have a major impact on highly infiltrative and/or rapidly growing gliomas. Thus, the precise way in which such cellular and microenviromental changes affect the overall invasive potential of tumours can be expected to depend on the specific intrinsic glioma cell features.

### Variations in oxygen consumption and vaso-occlusion produce opposing effects on glioma invasion

Figures 3(C,D) and 4(C,D) show simulation maps of the front speed and infiltration width for tumours characterised by different combinations of the intrinsic cell features *D* and *b*. In particular, these properties of tumour invasion are determined by a non-linear relationship between the effective diffusion *D*_eff_ and proliferation *b*_eff_ rates of glioma cells. For instance, in the simplest model corresponding to the classical Fisher-Kolmogorov equation (*Model I*), the tumour front speed is proportional to 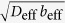 and the infiltration width to 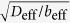. Model simulations suggest that, depending on the particular intrinsic tumour features, variations in the rates of glioma cell oxygen consumption and vaso-occlusion produce opposing effects on the resulting front speed and infiltration width. In fact, we found that there is a trade-off between tumour growth and invasion, which might be a reason why one-size-fits-all vaso-modulatory interventions are not effective enough to induce tumor clearance. These findings are counter-intuitive and might have important implications for the clinical application of modulatory interventions targeting glioma cell oxygen consumption and vascular occlusive events.

#### Cell oxygen consumption changes reveal a critical proliferation rate for glioma invasion

Analysis of the *Model II*, i.e. under the assumption of a constant density of functional tumour vasculature, reveals that variations in the rate at which glioma cells consume oxygen *h*_2_ produce opposing effects on the tumour front speed. More precisely, Fig. 3(C) shows that there exists a critical glioma cell proliferation rate b^*^ for which the front speed in tumours characterised by *b* > b^*^ decreases at higher values of *h*_2_, while on the contrary tumours with *b* < b^*^ invade faster displaying diffusely infiltrative growth patterns. Assuming that the tumour front speed is proportional to the product of effective diffusion and proliferation rates, we can readily explain the aforementioned simulation results for variations of *h*_2_. On one hand, in tumours with glioma cell proliferation rates *b* above the critical threshold b^*^, the effective migration and proliferation mechanisms compensate each other, leaving almost-invariant the speed of the invading front. On the other hand, in the cases of tumours with *b* < b^*^, while the effective proliferation rate is not significantly affected, the migratory activity of glioma cells is higher for increasing values of *h*_2_, which results in faster tumour front propagation speeds.

The flatness/steepness of the tumour front is proportional to a ratio of effective glioma cell diffusion and proliferation rates. When oxygen in the microenvironment is not limited, highly diffusive tumours evolve with large and flat fronts, whereas increased glioma cell proliferation results in short and steep fronts. However, under oxygen-limiting conditions the shape of the evolving tumour front is markedly influenced by the specific rate at which glioma cells consume oxygen. Figure 3(D) shows that variations in the rate of oxygen consumption produce the same overall effects on the infiltration width. Comparative simulation maps in Fig. 3(D) reveal that whatever the intrinsic glioma cell features, an arbitrary increase (decrease) in the oxygen consumption rate leads to more (less) invasive tumours. Indeed, the effective proliferation capacity of glioma cells is reduced due to increasing oxygen consumption rates, and in turn hypoxia-induced cell migration is enhanced, resulting in more aggressive, infiltrative tumour growth patterns.

#### Modulation of vaso-occlusion reveals a critical proliferation/diffusion ratio for glioma invasion

Simulations of the *Model III* reveal that for increasing vaso-occlusion rates *g*_2_, the tumour front speed is differently affected depending on the intrinsic diffusion and proliferation rates of glioma cells. In addition to the modulatory effects of oxygen availability on glioma growth and invasion, these processes are also influenced by vascularisation mechanisms. Comparative simulation maps in Fig. 4(C) evidence that in tumours with the intrinsic cell features *D* and *b* inside a region delimited by a critical rate *b*^+^ and an approximate ratio between diffusion and proliferation rates Λ^+^ = *b*/*D*, the invading front moves faster as *g*_2_ increases. Besides, the front speed slightly decreases or remains almost constant in the rest of tumours, i.e. with parameter values of *D* and *b* outside of such region. In particular, tumours characterised by *b* < *b*^+^ evolve at low cellular density and thus vascular occlusive events due to increased mechanical pressure by glioma cells hardly occur. On the other hand, increasing vaso-occlusion rates in tumours with *b* > *b*^+^ enhances the effective cell migration towards better vascularised brain tissue regions. Although vascular occlusion limits the proliferative activity of glioma cells, faster tumour front speeds are predicted as long as the triggered migratory activity dominates over cell proliferation.

The infiltration width in tumours with *b* < *b*^+^ is almost unaffected for increasing vaso-occlusion rates as shown in Fig. 4(D). However, tumours characterised by *b* > *b*^+^ are also separated by an approximated linear relation between *D* and *b* with respect to variations in the infiltration width. In particular, more occlusion of the blood vessel results in larger flat fronts in tumours with cell proliferation/diffusion ratios above the critical value Λ^+^ for *b* > *b*^+^, while the infiltration width is reduced in the rest of tumours.

## Discussion

**Figure 5:**
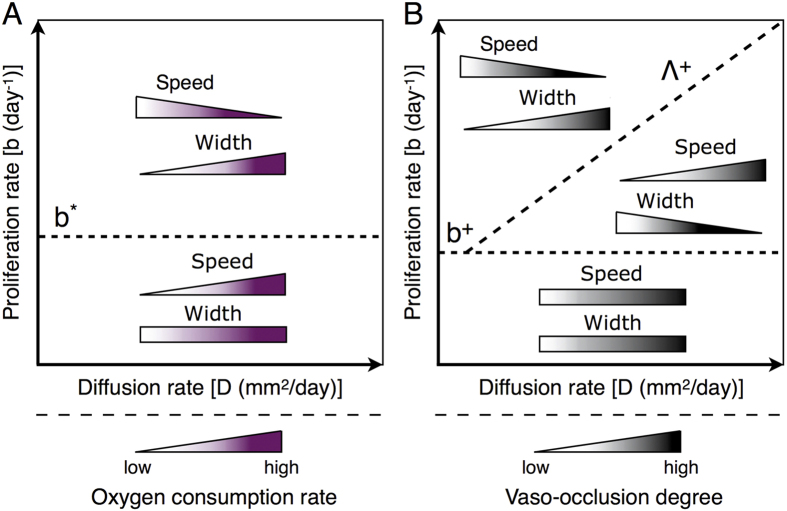
An overview of model simulation results. (**A**) Variations in the glioma cell oxygen consumption rate, under the assumption of constant functional vascularisation, reveal a critical proliferation rate b^*^ that separates tumour invasive behaviors in different regimes (*Model II*). (**B**) Variations in the vaso-occlusion rate reveal a critical proliferation/diffusion ratio Λ^+^ = *b*/*D* for *b* > *b*^+^ that separates tumour invasive behaviors in different regimes (*Model III*). Colour gradients from low to high represent the increase of glioma cell oxygen consumption and vaso-occlusion. The purple and black wedges/bars represent the corresponding effects on the tumour front speed and infiltration width for increasing/decreasing glioma cell oxygen consumption and vaso-occlusion rates.

In this work, we proposed a deterministic mathematical model of glioma growth and invasion that is formulated as a system of reaction-diffusion partial differential equations. Our glioma-vasculature interplay model accounts for the dynamics of normoxic and hypoxic glioma cells based on the Go-or-Grow mechanism which is in turn influenced by the functional tumour vasculature and the concentration of oxygen in the microenvironment. In particular, we focused on the effect of variations in the glioma cell oxygen consumption and vascular occlusion on prognostically-relevant characteristics of tumour invasion, i.e. the front speed and infiltration width. The main model results are summarised in Fig. 5.

The model analysis revealed that increasing glioma cell oxygen consumption and vaso-occlusion rates results in more diffusive and less proliferative tumours. In both scenarios, the average oxygen concentration in the tumour microenvironment decreases, which limits glioma cell proliferation and enhances hypoxia-induced migration. This is in line with previous clinical and histopathological observations that hypoxia strongly correlates with glioma malignancy^7^, as well as triggers tumour cell migration towards better oxygenated regions leading to pseudopalisade formation^19^,^20^,^21^,^23^. However, the extent to which such oxygen-mediated cell responses to blood vessel occlusion influence glioma invasion depends on the specific intrinsic tumour features. Variations in the vaso-occlusion rate evidenced the existence of a critical ratio between diffusion and proliferation rates that separates glioma invasive behaviours in different regimes, see Fig. 5(B). This result is obtained for tumours characterised by sufficiently high cellular proliferation rates in which variations in the oxygen concentration, due to vascular occlusion or normalisation, significantly influence glioma cell dynamics. In such cases, variations in the vascular function are predicted to produce opposing effects on the tumour front speed and infiltration width. Moreover, we found that depending on the intrinsic tumour features two distinct regimes are identified, where the glioma invasive behaviour in response to vaso-modulatory interventions is completly different. A pro-thrombotic treatment is predicted to increase the front speed, but in turn reduces the infiltration capacity, of tumours characterised by a cell proliferation/diffusion ratio below the critical threshold. On the contrary, tumours in the other parameter regime, and under the same vaso-modulatory strategy, become increasingly infiltrative and slowly growing. Analogously, vascular normalisation is predicted to induce opposing effects on glioma invasion for the corresponding parameter regimes.

Recently, it has been shown that the migration/proliferation dichotomy can introduce a critical threshold on the glioma cell density that separates tumour growth and extinction dynamics, a phenomenon called Allee effect^14^. Interestingly, we also found critical parameter values that distinguish between different glioma invasive patterns with respect to variations in the cell oxygen consumption and vaso-occlusion rates. This is an emergent consequence of the Go-or-Grow plasticity, since in its absence (*Model I*) critical behaviours are not observed. Assuming or not tumour vascularisation dynamics, the Go-or-Grow induced criticality is expressed either in the form of a proliferation/diffusion ratio Λ^+^ = *b*/*D* for *b* > *b*^+^ or a critical proliferation rate b^*^ of glioma cells, respectively. More precisely, the critical thresholds b^*^ and Λ^+^ for *b* > *b*^+^ separate tumour behaviours in regimes where the front speed and infiltration width are differently affected by changes in the glioma cell oxygen consumption and vaso-occlusion rates. These findings highlight the importance of further investigating the therapeutic potential of targeting the Go-or-Grow phenomenon as a strategy to reduce glioma cell migration. Based on our model results, we can argue that *one-size-fits-all* vaso-modulatory interventions should be expected to fail to control glioma invasion due to the complexity of the mechanisms involved and inter-patient heterogeneity. This study supports the value of personalised medicine and provides a simplified, but useful modelling framework with predictive potential based on a precise tumour profiling from possible biopsy measurements and medical imaging. In particular, patient-based estimation of tumour cell proliferation and diffusion rates would be crucial components of such future tailored approaches to individualise treatment selection for glioma patients.

We believe that this work substantially expands the theoretical concepts of the invasive behavior of gliomas, suggesting that any vasculature-targeting therapeutic intervention will inevitably lead to a trade-off between the tumour front speed and infiltration width. This result suggests that vaso-modulatory interventions should be embedded in a personalised combination of different treatment protocols, in which anti-angiogenesis might be integrated with individually adjusted strategies targeting cell proliferation, metabolic transformation or immune responses. For instance, in the case of gliomas characterised by a cell proliferation/diffusion ratio above Λ^+^ = *b*/*D* for *b* > *b*^+^, a pro-thrombotic or an anti-vasogenic therapeutic technique may reduce the tumour front speed but at the same time leads to highly infiltrative behaviours, which makes this treatment strategy rather inappropriate. However, normalisation of the tumour blood vessels may result in faster growing gliomas with compact, less invasive morphologies. Thus, surgical resection could be considered to remove such compact tumours. In turn, the benefits of conventional treatment modalities such as chemo-, radio- and immunotherapy might significantly increase in well-vascularised and therefore normally oxygenated tumours^24^,^30^,^31^,^32^. Thus, an accurate tumour patient stratification during clinical decision-making is crucial for the efficacy of vasculature-targeting therapies, either inducing tumour blood vessel deterioration or normalisation.

We conclude by pointing out a number of related future research directions, as well as discussing some limitations of this work. Although in our model the vaso-occlusion term in equation (15) is rather phenomenological and more accurate modelling might be required, we think that these *in silico* findings provide new insights into the impact of functional vascular changes on glioma invasion. Furthermore, the migration/proliferation dichotomy of glioma cells has been modelled in the simplest possible way and more informed formulations depending on other tumour-related factors should be considered. In turn, intratumoral genetic diversity is not directly considered, but instead we take into account phenotypic diversity depending on oxygen availability, which has long been recognized as an important therapeutic factor. The latter is supported by evidence that genetic diversity is tumour-subtype specific and not significantly affected during treatment, while phenotypic heterogeneity is significantly different before and after therapy^73^. For simplicity, we carried out simulations in one spatial dimension but the model analysis can be extended to higher dimensions. Qualitative deviations from the one-dimensional case can only be expected if the model’s radial symmetry breaks down via an interface instability. In a two-dimensional continuous version of the Go-or-Grow model no interface instability was observed^74^, i.e. the system grows in a radially symmetric way. Although, our system involves additional external fields such as the functional tumour vasculature, preliminary results have shown no qualitative deviations from the one-dimensional case for a continuous vascular field. Despite the fact that our model involves a large number of parameters, their values were selected independently from each other based on published experimental data. For those parameters estimated, we verified that variations in their values do not affect the general conclusions of this study. At this stage, we restrict the modelling strategy to investigate the effects of vasculature-targeting interventions on glioma invasion, however we are aware that further cell intrinsic and extrinsic factors may play a crucial role. In fact, we also intend to explore the interactions between glioma and immune cells influenced by vascularisation mechanisms as an additional level of complexity given the potential benefits of immunomodulatory therapies^42^,^43^. In particular, tumour-associated macrophages are plastic cells involved in relevant mechanisms such as angiogenesis and cell migration, that can exhibit protumour phenotypes promoting immune evasion and metastasis. Therefore, modelling the dynamics and function of macrophages in tumour progression may highlight new targets to develop more effective therapies, which is particularly relevant in the light of recent advances in the molecular classification of gliomas^75^. We strongly believe that mathematical modelling offers a useful integrative approach for conventional radiological, biopsy and molecular tumour characterisation, potentially allowing for the prediction of treatment outcomes and translation into the clinical decision-making process.

## Additional Information

**How to cite this article**: Alfonso, J. C. L. *et al*. Why *one-size-fits-all* vaso-modulatory interventions fail to control glioma invasion: *in silico* insights. *Sci. Rep.* **6**, 37283; doi: 10.1038/srep37283 (2016).

**Publisher's note:** Springer Nature remains neutral with regard to jurisdictional claims in published maps and institutional affiliations.

## Supplementary Material

Supplementary Material

## Figures and Tables

**Table 1:** Parameter values considered in the model simulations (see the Supporting Material for further details).

Parameter	Description	Value	Source
Glioma Cells			
*D*	Intrinsic diffusion rate of glioma cells	[2.73 × 10^−3^, 2.73 × 10^−1^] mm^2^ day^−1^	16,55,76
*b*	Intrinsic proliferation rate of glioma cells	[2.73 × 10^−4^, 2.73 × 10^−2^] day^−1^	16,55,76
*N*	Brain tissue carrying capacity	10^2^ cells mm^−1^	77,78
*σ*_0_	Physiological oxygen concentration	1.0 nmol mm^−1^	79,80
*λ*_1_	Phenotypic switching parameter^(†)^	2.0 nmol mm^−1^	*Model specific*
*λ*_2_	Phenotypic switching parameter^(‡)^	{0.5, 1.0, 2.0}	*Model specific*
Oxygen			
*D*_*σ*_	Diffusion rate of oxygen	1.51 × 10^2^ mm^2^ day^−1^	22,81,82
*h*_1_	Oxygen supply rate	3.37 × 10^−1^ day^−1^	83, 84, 85
*h*_2_	Glioma cell oxygen consumption rate	[5.73 × 10^−3^, 1.14 × 10^−1^] mm cell^−1^ day^−1^	68,86
Vasculature			
*D*_*v*_	Vasculature dispersal rate	5.0 × 10^−4^ mm^2^ day^−1^	16,22,87
*g*_1_	Vasculature formation rate	10^−1^ day^−1^	22,88,89
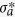	Oxygen concentration threshold for hypoxia	2.5 × 10^−1^ nmol mm^−1^	82,90,91
*K*	Half-maximal pro-angiogenic factor concentration	1.0 nmol mm^−1^	*Estimated*
*g*_2_	Vaso-occlusion rate	[5.0 × 10^−13^, 1.5 × 10^−11^] cell^−*n*^ mm^*n*^ day^−1^	*Estimated*
*n*	Dimensionless vaso-occlusion degree	6	*Estimated*

^(†)^Proliferative to migratory. ^(‡)^Migratory to proliferative.
