# Supplementary material for: Why one-size-fits-all vaso-modulatory interventions fail to control glioma invasion: in silico insights
Source: Sci Rep. 2016 Nov 23;6:37283. doi: 10.1038/srep37283 (PMC5120360; doi:10.1038/srep37283)
Supplement: Supplementary Material [file srep37283-s1.pdf]

Why *one-size-fits-all* vaso-modulatory interventions fail to control glioma invasion: *in silico* insights

J. C. L. Alfonso[1,2], A. Köhn-Luque[1,3], T. Stylianopoulos[4], F. Feuerhake[5,6], A. Deutsch[1] and H. Hatzikirou[2,1,†]

(1) Center for Information Services and High Performance Computing, Technische Universität Dresden, 01062 Dresden, Germany.

(2) Braunschweig Integrated Centre of Systems Biology, Helmholtz Center for Infectious Research, 38124 Braunschweig, Germany.

(3) Institute of Basic Medical Sciences, Faculty of Medicine, University of Oslo, 0317 Oslo, Norway.

(4) Cancer Biophysics Laboratory, University of Cyprus, 1678 Nicosia, Cyprus.

(5) Institute of Pathology, Medical School of Hannover, 30625 Hannover, Germany.

(6) Institute of Neuropathology, University Clinic Freiburg, 79117 Freiburg, Germany.

† Corresponding author: haralampos.hatzikirou@helmholtz-hzi.de

The authors have declared that no competing interest exists.

Supplementary Material

1.1 Model implementation

Numerical solutions of the proposed glioma-vasculature interplay model were obtained by implementing the finite element method and the backward Euler scheme for spatial and temporal discretisations, respectively [1,2]. The system of coupled partial differential equations (14)-(16) was first transformed into a weak formulation, which resulted in a system of ordinary differential equations with respect to time. The one-dimensional domain of simulation was divided into a finite number of distinct and non-overlapping segments. The integrals involved in the weak form of the system were calculated on each domain element by means of a Gaussian quadrature formula, which exactly integrates the resulting polynomials [3]. The backward Euler scheme was then used to obtain the temporal discretisation that resulted in a nonlinear system of equations solved at each instant of time by the Newton-Raphson method [1]. The model was implemented using MATLAB R2012a (The MathWorks Inc., Natick, USA - *www.mathworks.com*) and simulations were carried out in a SuSE Linux Enterprise Server 11 with 5888 core AMD Opteron 6274 2.2GHz, 92 nodes each with 64 cores and 64 to 512 GB of memory.

1.2 Model simulation domain

The system of equations (14)-(16) was solved in a one-dimensional domain Ω of length $L = 200$ mm for a total simulation time of about 3 years, i.e. $T_f = 1095$ days. The independent system variables are the time t and space x with $0 \leq x \leq L$ and $0 \leq t \leq T_f$. The x-axis can be thought of as a two-dimensional domain which is spatially averaged in one direction. The simulation domain, either inside the region occupied by glioma cells or outside representing the normal brain tissue, was discretised into an irregular grid varying the segment lengths from 2.5×10^{-3} mm to 2.5×10^{-2} mm. The time step was taken equal to 0.25 day, i.e. about 6 hours. Both, the segment length and the time step were properly selected to ensure numerical stability.

1.3 Model observables

We characterised the process of glioma invasion by the tumour front speed and infiltration width, see Figure S1. The front speed was estimated by the change rate of the point of maximum slope in $\rho(x,t)$ at the end of simulations T_f. In turn, the infiltration width was defined by the difference between the points where glioma cell density is 80% and 2% of the maximum cellular density at simulation time T_f.

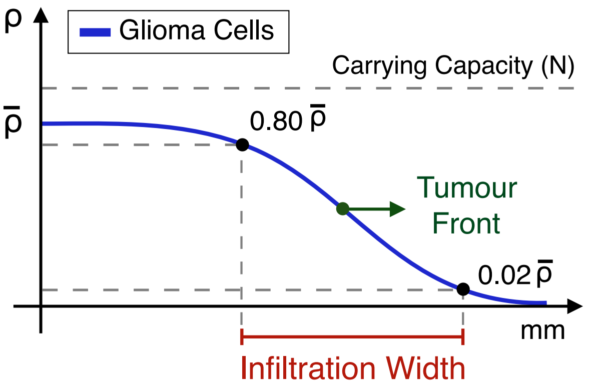

Figure S1: Model observables. The tumour front (green) is defined by the point of maximum slope in $\rho(x,t)$, and the infiltration width (red) by the points where glioma cell density is 80% and 2% of $\overline{\rho}$.

2 Model parameterisation

2.1 Initial conditions

Density of the functional tumour vasculature and the oxygen concentration were initialised in the simulation domain Ω as $v_0 = 1/2$ and $\sigma_0 = 1.0$ nmol mm^{-1}, respectively. In turn, the initial density of glioma cells was given by the product of $p_0 = 40$ cells mm^{-1} with a continuous approximation of the Heaviside decreasing step function $\tilde{H}_\gamma(x-\epsilon) = 1 - \left(1/\left(1 + e^{-2\gamma(x-\epsilon)}\right)\right)$ for $x \in \Omega$ with $\gamma = 1.0 \times 10^1$ and $\epsilon = 0.5$. The latter parameter choice provided continuity on the initial conditions, as well as guaranteed numerical stability. At both extremes of the simulation domain Ω, no-flux boundary conditions were imposed.

2.2 Density of glioma cells, $\rho(x,t)$

- Intrinsic diffusion rate of glioma cells D (in mm^2 day^{-1}). Several theoretical and image-based studies using a data-driven Fisher-Kolmogorov model suggest that the diffusion rate of glioma cells is a patient-specific parameter [4–8]. Estimates of D have been reported to vary from 2.73×10^{-3} to 2.73×10^{-1} mm^2 day^{-1}, which is supposed to cover the range from low- to high-grade gliomas [4,5,8,9]. Notice that $D_\rho = D/\alpha(\sigma_0)$, see equation (4) where $\alpha(\sigma)$ is defined.

- Intrinsic proliferation rate of glioma cells b (in day^{-1}). Similar experimental measurements, as reported for the diffusion rate of glioma cells D, evidence that the proliferation rate b is also a patient-specific parameter [4–8]. Estimates of b have been reported to vary from 2.73×10^{-4} to 2.73×10^{-2} day^{-1}, which is supposed to cover the range from low- to high-grade gliomas [4,5,8,9]. Notice that $b_\rho = b/\beta(\sigma_0)$, see equation (5) where $\beta(\sigma)$ is defined.

- Brain tissue carrying capacity N (in cells mm^{-1}). This parameter represents the limiting concentration of glioma cells that a domain element (normal brain tissue) can hold. Considering the average glioma cell diameter of about 10 μm [8], the one-dimensional carrying capacity is about 10^2 cells mm^{-1}.

Notice that this estimate of N has been considered in previous models of brain tumour growth and invasion [10, 11].

- Physiological oxygen concentration in the normal brain tissue σ_0 (in nmol mm^{-1}). Although *in vivo* estimates of oxygen tension P_{O2} in the normal brain tissue may vary with respect to measurement methods and several other factors, a suitable experimental value for σ_0 is 40 mmHg [12–15]. The Henry's law [16] was then used to obtain the physiological oxygen concentration as follows

$$\sigma_0 = 40/k_H \approx 2.068 \times 10^{-9} \text{ mol mm}^{-3} = 2.068 \text{ nmol mm}^{-3},$$

where $k_H \approx 1.934 \times 10^{10}$ mm^3 mmHg mol^{-1} is the Henry's law constant for oxygen at normal body temperature 37 °C.

To convert the above three-dimensional estimate of the oxygen concentration into its equivalent one-dimensional concentration, we multiplied by the area of a transversal tumour section. We assumed that such section is equivalent to the surface area of a sphere of radius r, where $A = 4\pi r^2$. Moreover, we considerd that $r = 200$ μm $= 2.0 \times 10^{-1}$ mm is the characteristic nutrient diffusion length, which is consistent with the experimentally observed thickness of viable rims of tumour cells in spheroids [17–19]. Then, $A = 4\pi(2 \times 10^{-1})^2 = 16\pi \times 10^{-2}$ mm^2 and we have that

$$\sigma_0 = 2.068 \text{ nmol mm}^{-3} \cdot (16\pi \cdot 10^{-2} \text{ mm}^2) \approx 1.0 \text{ nmol mm}^{-1}.$$

- Phenotypic switching parameter (proliferative to migratory) λ_1 (in nmol mm^{-1}). We assumed that $\lambda_1 = \sigma_M$ in the phenotypic switching function of glioma cells $f_{21} = \lambda_1 - \sigma$, where σ_M is the maximum oxygen concentration in the brain tissue. Oxygen tension P_{O2} in the normal brain tissue has been estimated to range from 10 to 80 mmHg [20, 21]. Accordingly, we considered that $\sigma_M = 2.0$ nmol mm^{-1}, i.e. for an oxygen tension P_{O2} equal to 80 mmHg, which is two times higher than the physiological oxygen concentration σ_0.

- Phenotypic switching parameter (migratory to proliferative) λ_2 (dimensionless). The effects of λ_2 on glioma growth and invasion was investigated by considering the overall effective proliferation rate of glioma cells given by

$$B = b\,\frac{\beta(\sigma)}{\beta(\sigma_0)} = b\,\frac{(\lambda_2 - 1)\sigma_0 + \lambda_1}{(\lambda_2 - 1)\sigma + \lambda_1}\,\frac{\sigma}{\sigma_0},$$

where, taking into account that $\lambda_1 = \sigma_M$, we can distinguish three representative cases:

(i) If $0 < \lambda_2 < 1$, then $B = b\,\dfrac{\sigma_M - |\lambda_2 - 1|\sigma_0}{\sigma_M - |\lambda_2 - 1|\sigma} \cdot \dfrac{\sigma}{\sigma_0} \propto \dfrac{\sigma}{\dfrac{\sigma_M}{|\lambda_2 - 1|} - \sigma}$.

(ii) If $\lambda_2 = 1$, then $B = b\,\dfrac{\sigma}{\sigma_0} \propto \sigma$.

(iii) If $\lambda_2 > 1$, then $B = b\,\dfrac{|\lambda_2 - 1|\sigma_0 + \sigma_M}{|\lambda_2 - 1|\sigma + \sigma_M} \cdot \dfrac{\sigma}{\sigma_0} \propto \dfrac{\sigma}{\dfrac{\sigma_M}{|\lambda_2 - 1|} + \sigma}$.

According to the cases (i)-(iii), we can reduce the model analysis to the following three parameter values: $\lambda_2 = \{0.5,\ 1.0,\ 2.0\}$, see Figure S2. Notice that in the limiting case of $\lambda_2 = 0$ glioma cells do not proliferate, and therefore we neglected this scenario. Although model simulations were obtained for the phenotypic switching parameter $\lambda_2 = 1.0$, we investigate in Section 3 below the effects of λ_2 variations on the invasive behaviour of gliomas, see Figures S4 and S5.

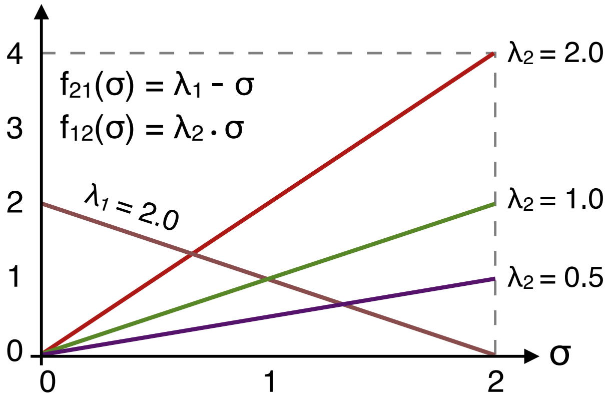

Figure S2: Oxygen-dependent phenotypic switching functions of glioma cells based on the migration/proliferation dichotomy.

2.3 Oxygen concentration, $\sigma(x,t)$

- Diffusion rate of oxygen D_σ (in mm^2 day^{-1}). Based on experimental data, the oxygen diffusion rate in tumour tissues at 37 °C has been reported equal to 1.75×10^{-5} cm^2 s^{-1} [22]. Accordingly, we considered that $D_\sigma = 1.51 \times 10^2$ mm^2 day^{-1}, which is in agreement with previous estimates of the oxygen diffusion rate [23–25].

- Oxygen supply rate h_1 (in day^{-1}). Experimental estimates of the transvascular permeability to oxygen Pm_{O2} have been reported in the range of 3×10^{-5} to 3×10^{-4} m s^{-1} [26]. In turn, the ratio of capillary surface area to volume $\frac{S}{V}$ has been observed to vary from 0.13 to 0.33 m^{-1} [26]. Thus, $Pm_{O2} \cdot \frac{S}{V}$ lies in the range of 4.0×10^{-6} to 1.0×10^{-4} s^{-1}, which is equivalent to the oxygen supply rate h_1 in equation (16) [26]. These estimates are in line with other oxygen supply rates reported [27,28], i.e. $h_1 = 3.5 \times 10^{-6}$ s^{-1} and 4.0×10^{-6} s^{-1}. Accordingly, we considered that $h_1 = 3.37 \times 10^{-1}$ day^{-1}, which is in the range of oxygen supply rates above.

- Oxygen consumption rate h_2 (in mm cell^{-1} day^{-1}). The rate at which glioma cell consume oxygen has been reported to vary from 2 to 40 μl g^{-1} min^{-1} [29, 30]. Considering the average mass of a cancer cell equal to 10^{-9} kg [25], and taking into account that 1 μl = 1 mm^3, we have that h_2 lies in the range of 2.88×10^{-3} to 5.76×10^{-2} mm^3 cells^{-1} day^{-1}. As explained above for the physiological oxygen concentration σ_0, we converted the three-dimensional estimate of the oxygen consumption rate into its equivalent one-dimensional rate dividing by the area of a transversal tumour section equal to $A = 16\pi \cdot 10^{-2}$ mm^2. We have then that h_2 varies from 5.73×10^{-3} to 1.14×10^{-1} mm cell^{-1} day^{-1}.

2.4 Density of functional tumour vasculature, $v(x,t)$

- Vasculature dispersal rate D_v (in mm^2 day^{-1}). Experimental estimates of endothelial cell motility rate in different microenvironmental conditions have been reported in the range of 10^{-3} to 10^{-4} mm^2 day^{-1} [31, 32]. Accordingly, we considered that $D_v = 5.0 \times 10^{-4}$ mm^2 day^{-1}, which has been considered in previous models of vascularised tumour growth [8, 24, 33].

- Vasculature formation rate g_1 (in day^{-1}). We take $g_1 = 1.0 \times 10^{-1}$ day^{-1} by assuming that new tumour blood vessels are formed in a timescale of hours [24,34,35]. We remark that g_1 variations change the model results only quantitatively, while qualitative phenomena are conserved.

- Oxygen concentration threshold for hypoxia σ_a^* (in nmol mm^{-1}). Although no consensus exists regarding hypoxic thresholds, tissues with oxygen tension P_{O2} below 10 mmHg are usually considered under hypoxia [25, 36, 37]. Indeed, tissues with P_{O2} in the range of 5.0 to 7.5 mmHg are supposed under moderate hypoxia, while less than or equal to 2.5 mmHg under severe hypoxia [36]. Accordingly, we assumed that $\sigma_a^* = 2.5 \times 10^{-1}$ nmol mm^{-1}, see also the derivation of the physiological oxygen concentration in the normal brain tissue σ_0 for further details.

- Half-maximal pro-angiogenic factor concentration K (in nmol mm^{-1}). We assumed that the natural decay rate of pro-angiogenic factors is much smaller than the consumption rate by endothelial cells forming the vascular network, i.e. $k_3 \ll k_2$ in equation (8) [38]. Therefore, taking into account the quasi-steady state approximation of equation (8) for the pro-angiogenic factor concentration $a(x,t)$ given by

$$a = \frac{k_1\,\rho\,\tilde{\mathrm{H}}_\theta(\sigma - \sigma_a^*)}{k_2 v + k_3},$$

we have that the Michaelis-Menten kinetics of the density of functional tumour vasculature $v(x,t)$ in equation (11) is as follows

$$g_1\frac{a}{\mu + a} = g_1\frac{\dfrac{k_1\rho\tilde{\mathrm{H}}_\theta(\sigma-\sigma_a^*)}{k_2 v + k_3}}{\mu + \dfrac{k_1\rho\tilde{\mathrm{H}}_\theta(\sigma-\sigma_a^*)}{k_2 v + k_3}} = g_1\frac{\dfrac{k_1}{k_2}\dfrac{\rho}{v}\tilde{\mathrm{H}}_\theta(\sigma-\sigma_a^*)}{\mu + \dfrac{k_1}{k_2}\dfrac{\rho}{v}\tilde{\mathrm{H}}_\theta(\sigma-\sigma_a^*)} = g_1\frac{\dfrac{\rho}{v}\tilde{\mathrm{H}}_\theta(\sigma-\sigma_a^*)}{K + \dfrac{\rho}{v}\tilde{\mathrm{H}}_\theta(\sigma-\sigma_a^*)},$$

where the temporal t and spatial x coordinates in the arguments of variables have been omitted for notational simplicity. Notice that $K = \mu k_2/k_1$ is a positive parameter denoting the concentration of pro-angiogenic factors at which the formation rate of functional tumour vasculature is half-maximal. $\tilde{\mathrm{H}}_\theta(\sigma - \sigma_a^*)$ is a continuous approximation of the Heaviside decreasing step function $H(\xi)$, defined as $H(\xi) = 1$ if $\xi \leq 0$ and $H(\xi) = 0$ if $\xi > 0$, given by

$$\tilde{\mathrm{H}}_\theta(\sigma - \sigma_a^*) = 1 - \frac{1}{1 + e^{-2\theta(\sigma-\sigma_a^*)}},$$

where $\theta = 1.0 \times 10^1$ and $K = 1.0 \times 10^1$ nmol mm^{-1}. We remark that variations of K slightly change the model results quantitatively, while qualitative phenomena are conserved.

- Vaso-occlusion term $G(v,\rho) = g_2 v \rho^n$. Figure S3(A) shows a schematic representation of vaso-occlusion, see equation (15). We assumed from experimental data that occlusion/collapse of tumour blood vessels only occurs for glioma cell densities greater than $N/2$, where N is the brain tissue carrying capacity [39]. Accordingly, we can distinguish two representative cases:

$$\text{(i) If } \rho \leq N/2, \quad \text{then} \quad G(v,\rho) = g_2 v \rho^n = g_2\frac{N^n}{2^{n+1}} \approx 0.$$

$$\text{(ii) If } \rho > N/2, \quad \text{then} \quad G(v,\rho) = g_2 v \rho^n > g_2\frac{N^n}{2^{n+1}} > 0.$$

Then, by considering the functional tumour vasculature at normal density, i.e. $v = 1/2$, we have that to satisfy the assumption above, low and high values of g_2 and n are required, respectively. Accordingly, we fixed $n = 6$, and investigated the effects of different vaso-occlusion rates $g_2 = \{5.0 \times 10^{-13},\ 5.0 \times 10^{-12},\ 1.5 \times 10^{-11}\}$ cell^{-n} mm^n day^{-1} on the effective invasive behaviour of gliomas. Notice that lower values of n do not allow to reproduce the experimental observation that vaso-occlusion occurs at tumour cell densities greater than $N/2$ [39].

Figure S3(B) illustrates the dependence of the vaso-occlusion term $G(v,\rho)$ in equation (15) on the density of glioma cells $\rho(x,t)$ for $n = 6$, $v = 1/2$ and the g_2 values considered. In turn, Figure S3(C) shows

simulation maps of the vaso-occlusion percentage depending on the intrinsic diffusion D and proliferation b rates of glioma cells at the end of simulations $T_f = 3$ years. This percentage of occluded tumour vasculature was obtained as the ratio between the integral of $v(x, T_f)$, from $x = 0$ to the point x_v where $v = 1/2$, and the area of the rectangle $\frac{1}{2}x_v$, see Figure S3(A). We found that vaso-occlusion increases with the proliferation rate of glioma cells b, see Figure S3(C).

The term $G(v, \rho) = g_2 v \rho^n$ in equation (15) was selected to model vaso-occlusion based on our experimental experience and theoretical arguments. In particular, extensive tumour blood vessel occlusion/collapse takes place when the solid stress exceeds a critical level [40, 41]. Prior to this critical stress threshold, blood vessel occlusion/collapse is moderate [41]. We remark that the use of a different expression for $G(v, \rho)$ would change the results only quantitatively, but it is not expected to affect the general conclusions of this study.

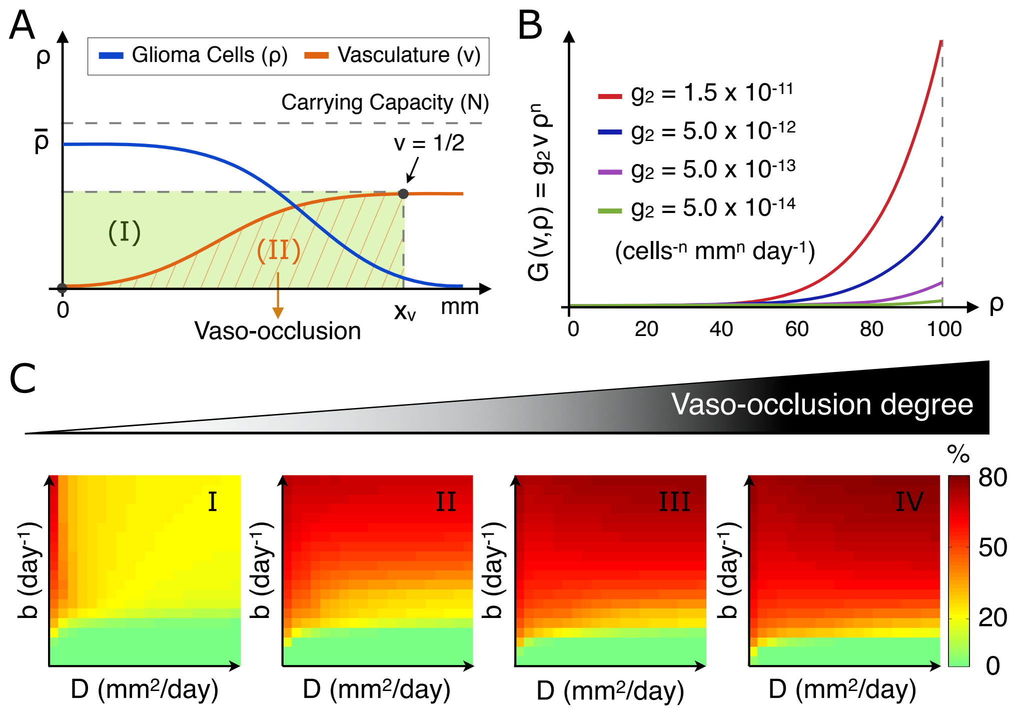

Figure S3: (A) Schematic representation of vaso-occlusion. (B) Dependence of the vaso-occlusion term $G(v, \rho) = g_2 v \rho^n$ in equation (15) on the density of glioma cells ρ for $v = 1/2$, $n = 6$ and different vaso-occlusion rates g_2. (C) Vaso-occlusion percentage, at the end of simulations $T_f = 3$ years, with respect to the intrinsic diffusion D and proliferation b rates of glioma cells for a constant oxygen consumption rate $h_2 = 5.73 \times 10^{-3}$ mm cell^{-1} day^{-1} and $g_2 = \{5.0 \times 10^{-14},\ 5.0 \times 10^{-13},\ 5.0 \times 10^{-12},\ 1.5 \times 10^{-11}\}$ cells^{-n} mm^n day^{-1} in simulation maps I-IV, respectively. The other parameters are as in Table 1.

Theoretical justification of the vaso-occlusion term $G(v, \rho) = g_2 v \rho^n$

A main reason for the appearance of blood vessel occlusion is the increased mechanical pressure exerted on them in regions of high tumour cell density [42]. We assumed that tumours behave as colloid fluids [43], where the pressure on such fluid can be calculated by means of the Viral theorem [44] as follows

$$\frac{P}{kT} = \rho + B_2 \rho^2 + B_3 \rho^3 + \dots \qquad \text{(S1)}$$

where k is the Boltzmann constant and T is the temperature. The second term of the expansion above is given by

$$B_2 = 2\pi \int_0^\infty [1 - g(r)]r^2 \, dr, \tag{S2}$$

where $g(r)$ is the radial distribution function, which depends on the spatial correlations of the fluid particles. Then, for $g(r) = 1$ the system of particles is decorrelated, and by means of equations (S1) and (S2) we have that the state equation for ideal gases is given by

$$\frac{P}{kT} = \rho \Rightarrow P = \rho kT. \tag{S3}$$

Then, assuming that B_i with $i = 2, 3$ are time-invariant in equation (S1), we can consider that

$$\frac{P}{kT} \propto \rho^n, \tag{S4}$$

where n is the constant associated to the maximum term B_n that depends on the exact interaction between particles / tumour cells.

3 Effect of the phenotypic switching parameter λ_2 on model observables

Model simulations in Figures (3) and (4) were obtained for the phenotypic switching parameter $\lambda_2 = 1.0$. Therefore, in order to complete the analysis of the proposed glioma-vasculature interplay model we investigated the effect of different values of $\lambda_2 = \{0.5,\ 1.0,\ 2.0\}$ on the invasive behaviour of gliomas. In particular, these λ_2 values include the three representative cases discussed above, see Figure S2. As shown in Figures S4 and S5, for increasing λ_2 values the tumour front speed increases, while the infiltration width decreases. Based on these results, we can argue that the invasive behaviour of gliomas in response to variations of λ_2 is only quantitatively affected, while qualitative phenomena are conserved.

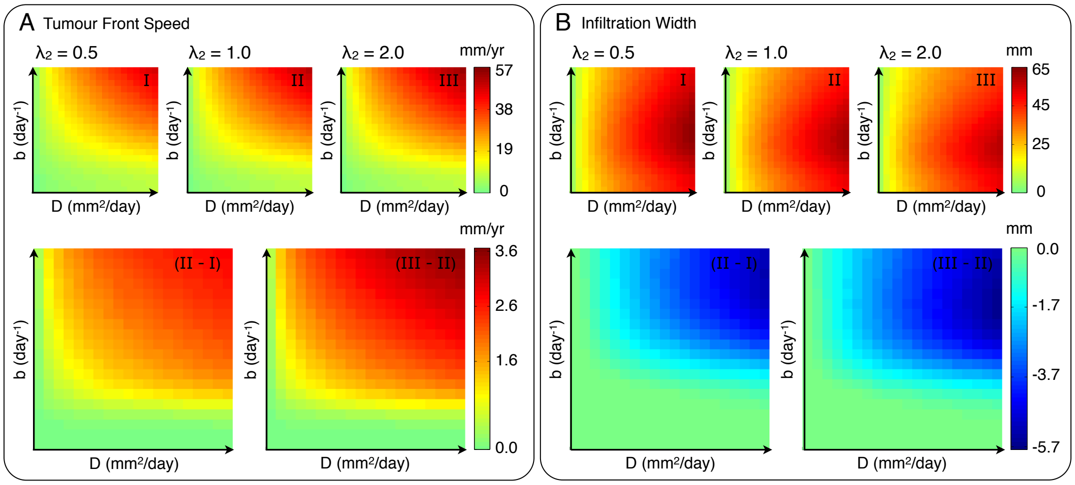

Figure S4: **Model observables with respect to parameter λ_2 for constant functional vascularisation (*Model II*).** Simulation maps with respect to the intrinsic proliferation $b \in [2.73 \times 10^{-4},\ 2.73 \times 10^{-2}]$ days^{-1} and diffusion $D \in [2.73 \times 10^{-3},\ 2.73 \times 10^{-1}]$ mm^2 days^{-1} rates of glioma cells. (A) Tumour front speed and (B) infiltration width for a constant glioma cell oxygen consumption rate $h_2 = 5.73 \times 10^{-3}$ mm cell^{-1} day^{-1}, and $\lambda_2 = \{0.5,\ 1.0,\ 2.0\}$ in simulation maps I-III, respectively. (A-B) Differences between the simulation maps I-III. The other parameters are as in Table 1.

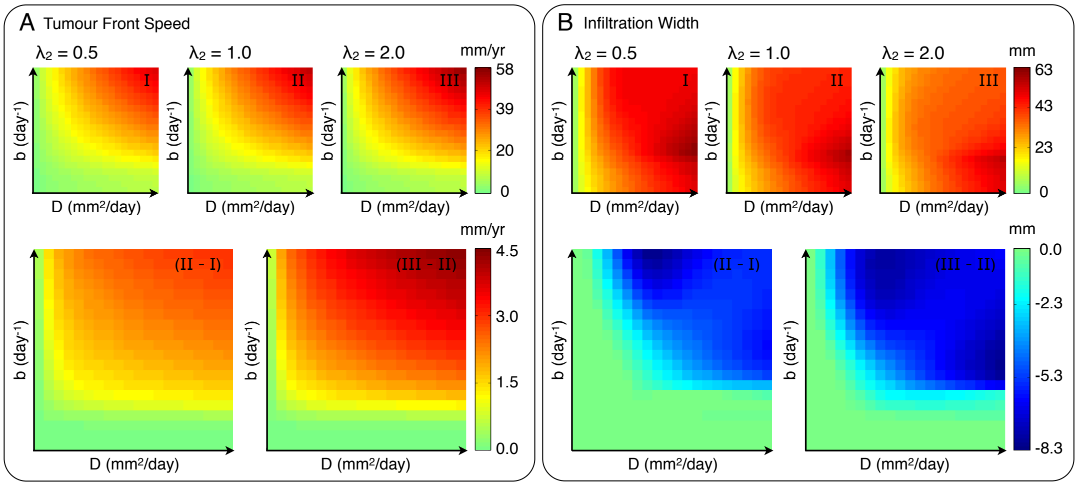

Figure S5: **Model observables with respect to parameter λ_2 (*Model III*).** Simulation maps with respect to the intrinsic proliferation $b \in [2.73 \times 10^{-4},\ 2.73 \times 10^{-2}]$ days^{-1} and diffusion $D \in [2.73 \times 10^{-3},\ 2.73 \times 10^{-1}]$ mm^2 days^{-1} rates of glioma cells. (A) Tumour front speed and (B) infiltration width for a constant glioma cell oxygen consumption $h_2 = 5.73 \times 10^{-3}$ mm cell^{-1} day^{-1} and vaso-occlusion $g_2 = 5.0 \times 10^{-12}$ cells^{-n} mm^n day^{-1} rates, and $\lambda_2 = \{0.5,\ 1.0,\ 2.0\}$ in simulation maps I-III, respectively. (A-B) Differences between the simulation maps I-III. The other parameters are as in Table 1.

References

[1] S. Larsson and V. Thomee, *Partial Differential Equations with Numerical Methods*. Texts in Applied Mathematics, Springer Berlin Heidelberg, 2008.

[2] C. Johnson, *Numerical Solution of Partial Differential Equations by the Finite Element Method*. Dover Books on Mathematics Series, Dover Publications, Incorporated, 2012.

[3] A. Khursheed, *The Finite Element Method in Charged Particle Optics*. The Springer International Series in Engineering and Computer Science, Springer US, 2012.

[4] H. Harpold, E. Alvord Jr, and K. Swanson, "The evolution of mathematical modeling of glioma proliferation and invasion," *Journal of Neuropathology & Experimental Neurology*, vol. 66, no. 1, pp. 1–9, 2007.

[5] K. Swanson, R. Rostomily, and E. Alvord Jr, "A mathematical modelling tool for predicting survival of individual patients following resection of glioblastoma: a proof of principle," *British Journal of Cancer*, vol. 98, no. 1, pp. 113–119, 2008.

[6] C. Wang, J. Rockhill, M. Mrugala, D. Peacock, A. Lai, K. Jusenius, J. Wardlaw, T. Cloughesy, A. Spence, R. Rockne, and et al., "Prognostic significance of growth kinetics in newly diagnosed glioblastomas revealed by serial imaging with a novel biomathematical model," *Cancer Research*, vol. 69, no. 23, pp. 9133–9140, 2009.

[7] R. Rockne, J. Rockhill, M. Mrugala, A. Spence, I. Kalet, K. Hendrickson, A. Lai, T. Cloughesy, E. Alvord Jr, and K. Swanson, "Predicting the efficacy of radiotherapy in individual glioblastoma patients in vivo: a mathematical modeling approach," *Physics in Medicine and Biology*, vol. 55, no. 12, p. 3271, 2010.

[8] K. Swanson, R. Rockne, J. Claridge, M. Chaplain, E. Alvord Jr, and A. Anderson, "Quantifying the role of angiogenesis in malignant progression of gliomas: in silico modeling integrates imaging and histology," *Cancer Research*, vol. 71, no. 24, pp. 7366–7375, 2011.

[9] M. Badoual, C. Gerin, C. Deroulers, B. Grammaticos, J. Llitjos, C. Oppenheim, P. Varlet, and J. Pallud, "Oedema-based model for diffuse low-grade gliomas: application to clinical cases under radiotherapy," *Cell Proliferation*, vol. 47, no. 4, pp. 369–380, 2014.

[10] S. Eikenberry, T. Sankar, M. Preul, E. Kostelich, C. Thalhauser, and Y. Kuang, "Virtual glioblastoma: growth, migration and treatment in a three-dimensional mathematical model," *Cell Proliferation*, vol. 42, no. 4, pp. 511–528, 2009.

[11] J. McDaniel, E. Kostelich, Y. Kuang, J. Nagy, M. Preul, N. Moore, and N. Matirosyan, “Data assimilation in brain tumor models,” in *Mathematical Methods and Models in Biomedicine*, pp. 233–262, Springer, 2013.

[12] A. Maas, W. Fleckenstein, D. de Jong, and H. van Santbrink, “Monitoring cerebral oxygenation: experimental studies and preliminary clinical results of continuous monitoring of cerebrospinal fluid and brain tissue oxygen tension,” in *Monitoring of Cerebral Blood Flow and Metabolism in Intensive Care*, pp. 50–57, Springer, 1993.

[13] J. Meixensberger, J. Dings, H. Kuhnigk, and K. Roosen, “Studies of tissue po2 in normal and pathological human brain cortex,” in *Monitoring of Cerebral Blood Flow and Metabolism in Intensive Care*, pp. 58–63, Springer, 1993.

[14] W. Hoffman, F. Charbel, G. Edelman, K. Hannigan, and J. Ausman, “Brain tissue oxygen pressure, carbon dioxide pressure and ph during ischemia.,” *Neurological Research*, vol. 18, no. 1, pp. 54–56, 1996.

[15] A. Carreau, B. Hafny-Rahbi, A. Matejuk, C. Grillon, and C. Kieda, “Why is the partial oxygen pressure of human tissues a crucial parameter? small molecules and hypoxia,” *Journal of Cellular and Molecular Medicine*, vol. 15, no. 6, pp. 1239–1253, 2011.

[16] W. Henry, “Experiments on the quantity of gases absorbed by water, at different temperatures, and under different pressures,” *Philosophical Transactions of the Royal Society of London*, pp. 29–276, 1803.

[17] H. Frieboes, X. Zheng, C. Sun, B. Tromberg, R. Gatenby, and V. Cristini, “An integrated computational/experimental model of tumor invasion,” *Cancer Research*, vol. 66, pp. 1597–1604, Feb. 2006.

[18] V. Cristini, H. Frieboes, X. Li, J. Lowengrub, P. Macklin, S. Sanga, S. Wise, and X. Zheng, “Nonlinear modeling and simulation of tumor growth,” in *Selected topics in cancer modeling: Genesis, evolution, immune competition, and therapy. Modelling and Simulation in Science, Engineering, and Technology* (N. Bellomo, M. Chaplain, and E. de Angelis, eds.), ch. 6, pp. 113–82, Boston, MA USA: Birkhäuser, 2008.

[19] H. Hatzikirou, J. Alfonso, S. Mühle, C. Stern, S. Weiss, and M. Meyer-Hermann, “Cancer therapeutic potential of combinatorial immuno-and vasomodulatory interventions,” *Journal of The Royal Society Interface*, vol. 12, no. 112, p. 20150439, 2015.

[20] K. Luoto, R. Kumareswaran, and R. Bristow, “Tumor hypoxia as a driving force in genetic instability,” *Genome Integr*, vol. 4, no. 5, pp. 10–1186, 2013.

[21] S. Crawford, “Is it time for a new paradigm for systemic cancer treatment? lessons from a century of cancer chemotherapy,” *Frontiers in Pharmacology*, vol. 4, no. 68, 2013.

[22] J. Grote, R. Susskind, and P. Vaupel, “Oxygen diffusivity in tumor tissue (ds-carcinosarcoma) under temperature conditions within the range of 20-40° c,” *Pflügers Archiv*, vol. 372, no. 1, pp. 37–42, 1977.

[23] A. Matzavinos, C. Kao, J. Green, A. Sutradhar, M. Miller, and A. Friedman, “Modeling oxygen transport in surgical tissue transfer,” *Proceedings of the National Academy of Sciences*, vol. 106, no. 29, pp. 12091–12096, 2009.

[24] I. Stamper, M. Owen, P. Maini, and H. Byrne, “Oscillatory dynamics in a model of vascular tumour growth-implications for chemotherapy,” *Biology Direct*, vol. 5, no. 1, p. 27, 2010.

[25] G. Powathil, M. Kohandel, M. Milosevic, and S. Sivaloganathan, “Modeling the spatial distribution of chronic tumor hypoxia: implications for experimental and clinical studies,” *Computational and Mathematical Methods in Medicine*, vol. 2012, 2012.

[26] C. Kelly and M. Brady, “A model to simulate tumour oxygenation and dynamic [18f]-fmiso pet data,” *Physics in Medicine and Biology*, vol. 51, no. 22, p. 5859, 2006.

[27] C. Eggleton, T. Roy, and A. Popel, “Predictions of capillary oxygen transport in the presence of fluorocarbon additives,” *American Journal of Physiology-Heart and Circulatory Physiology*, vol. 275, no. 6, pp. H2250–H2257, 1998.

[28] D. Goldman and A. Popel, “A computational study of the effect of capillary network anastomoses and tortuosity on oxygen transport,” *Journal of Theoretical Biology*, vol. 206, no. 2, pp. 181–194, 2000.

[29] P. Vaupel, F. Kallinowski, and P. Okunieff, “Blood flow, oxygen and nutrient supply, and metabolic microenvironment of human tumors: a review,” *Cancer Research*, vol. 49, no. 23, pp. 6449–6465, 1989.

[30] D. Grimes, C. Kelly, K. Bloch, and M. Partridge, “A method for estimating the oxygen consumption rate in multicellular tumour spheroids,” *Journal of The Royal Society Interface*, vol. 11, no. 92, p. 20131124, 2014.

[31] C. Stokes, D. Lauffenburger, and S. Williams, “Migration of individual microvessel endothelial cells: stochastic model and parameter measurement,” *Journal of Cell Science*, vol. 99, no. 2, pp. 419–430, 1991.

[32] S. Kouvroukoglou, K. Dee, R. Bizios, L. McIntire, and K. Zygourakis, “Endothelial cell migration on surfaces modified with immobilized adhesive peptides,” *Biomaterials*, vol. 21, no. 17, pp. 1725–1733, 2000.

[33] A. Anderson and M. Chaplain, “Continuous and discrete mathematical models of tumor-induced angiogenesis,” *Bulletin of Mathematical Biology*, vol. 60, no. 5, pp. 857–899, 1998.

[34] C. Shaifer, J. Huang, and P. Lin, “Glioblastoma cells incorporate into tumor vasculature and contribute to vascular radioresistance,” *International Journal of Cancer*, vol. 127, no. 9, pp. 2063–2075, 2010.

[35] M. Scianna, C. Bell, and L. Preziosi, “A review of mathematical models for the formation of vascular networks,” *Journal of Theoretical Biology*, vol. 333, pp. 174–209, 2013.

[36] L. Cárdenas-Navia, D. Yu, R. Braun, D. Brizel, T. Secomb, and M. Dewhirst, “Tumor-dependent kinetics of partial pressure of oxygen fluctuations during air and oxygen breathing,” *Cancer Research*, vol. 64, no. 17, pp. 6010–6017, 2004.

[37] P. Vaupel and A. Mayer, “Hypoxia in cancer: significance and impact on clinical outcome,” *Cancer and Metastasis Reviews*, vol. 26, no. 2, pp. 225–239, 2007.

[38] A. Köhn-Luque, W. de Back, Y. Yamaguchi, K. Yoshimura, M. Herrero, and T. Miura, “Dynamics of vegf matrix-retention in vascular network patterning,” *Physical Biology*, vol. 10, no. 6, p. 066007, 2013.

[39] T. Padera, B. Stoll, J. Tooredman, D. Capen, E. di Tomaso, and R. Jain, “Pathology: cancer cells compress intratumour vessels,” *Nature*, vol. 427, no. 6976, pp. 695–695, 2004.

[40] T. Stylianopoulos and R. Jain, “Combining two strategies to improve perfusion and drug delivery in solid tumors,” *Proceedings of the National Academy of Sciences*, vol. 110, no. 46, pp. 18632–18637, 2013.

[41] T. Stylianopoulos, J. Martin, M. Snuderl, F. Mpekris, S. Jain, and R. Jain, “Coevolution of solid stress and interstitial fluid pressure in tumors during progression: implications for vascular collapse,” *Cancer Research*, vol. 73, no. 13, pp. 3833–3841, 2013.

[42] D. Brat, A. Castellano-Sanchez, S. Hunter, M. Pecot, C. Cohen, E. Hammond, S. Devi, B. Kaur, and E. van Meir, “Pseudopalisades in glioblastoma are hypoxic, express extracellular matrix proteases, and are formed by an actively migrating cell population,” *Cancer Research*, vol. 64, no. 3, pp. 920–927, 2004.

[43] T. E. Angelini, E. Hannezo, X. Trepat, M. Marquez, J. J. Fredberg, and D. A. Weitz, “Glass-like dynamics of collective cell migration,” *Proceedings of the National Academy of Sciences*, vol. 108, no. 12, pp. 4714–4719, 2011.

[44] M. Kardar, *Statistical physics of particles*. Cambridge University Press, 2007.
